# Supplementary material for: Influence of the Crystal Forms of Calcium Carbonate on the Preparation and Characteristics of Indigo Carmine-Calcium Carbonate Lake
Source: Foods. 2024 Aug 20;13(16):2607. doi: 10.3390/foods13162607 (PMC11354058; doi:10.3390/foods13162607)
Supplement: Supplementary file 1 [file foods-13-02607-s001.zip › foods-3138246-supplementary.pdf]

Supplementary data

Influence of the crystal forms of calcium carbonate on the preparation and characteristics of indigo carmine-calcium carbonate lake

Table S1 Formulation details for preparing calcium carbonates and their colorant lakes.

Name	Na_2CO_3 (mol/L)	$CaCl_2$ (mol/L)	IC (mg)	Solvent	Volume after mixing (mL)
Calcite	0.2	0.2	0	Water	300
Calcite Lake (50 mg)	0.2	0.2	50	Water	300
Calcite Lake (200 mg)	0.2	0.2	200	Water	300
Calcite Lake (500 mg)	0.2	0.2	500	Water	300
Aragonite	0.2	0.2	0	Water	300
Aragonite Lake (50 mg)	0.2	0.2	50	Water	300
Aragonite Lake (200 mg)	0.2	0.2	200	Water	300
Aragonite Lake (500 mg)	0.2	0.2	500	Water	300
Vaterite	0.1	0.1	0	40% ethanol solution	300
Vaterite Lake (25 mg)	0.1	0.1	25	40% ethanol solution	300
Vaterite Lake (100 mg)	0.1	0.1	100	40% ethanol solution	300
Vaterite Lake (250 mg)	0.1	0.1	250	40% ethanol solution	300
ACC	0.1	0.1	0	Water	40
ACC Lake (3.3 mg)	0.1	0.1	3.3	Water	40
ACC Lake (13.3 mg)	0.1	0.1	13.3	Water	40
ACC Lake (33.3 mg)	0.1	0.1	33.3	Water	40

Table S2 The FTIR spectra peak assignments for calcium carbonates, colorant lakes and IC.

Samples	Wavenumber (cm^{-1})	Peak assignments	References
Calcite	876	The CO_3^{2-} out-of-plane deformation vibration	[20, 44-47]
	711	The C-O in-plane deformation vibration	
	1418	The C-O antisymmetric stretching vibration	
	1797	The stretching vibration of C=O	
Calcite Lake	876	The CO_3^{2-} out-of-plane deformation vibration	
	711	The C-O in-plane deformation vibration	
	1418	The C-O antisymmetric stretching vibration	
	1797	The stretching vibration of C=O	
	1643	The C=C pull-up on the benzene ring	
Aragonite	875	The CO_3^{2-} out-of-plane deformation vibration	
	714	The C-O in-plane deformation vibration	
	1084	The C-O stretching vibration	
	1495	The C-O antisymmetric stretching vibration	
	1786	The stretching vibration of C=O	
Aragonite Lake	856	The CO_3^{2-} out-of-plane deformation vibration	
	714	The C-O in-plane deformation vibration	
	1084	The C-O stretching vibration	
	1493	The C-O antisymmetric stretching vibration	
	1787	The stretching vibration of C=O	
Vaterite	879	The CO_3^{2-} out-of-plane deformation vibration	
	747	The C-O in-plane deformation vibration	
	1087	The C-O stretching vibration	
	1490	The C-O antisymmetric stretching vibration	
Vaterite Lake	879	The CO_3^{2-} out-of-plane deformation vibration	
	747	The C-O in-plane deformation vibration	
	1105	The C-O stretching vibration	
	1491	The C-O antisymmetric stretching vibration	
	1643	The C=C pull-up on the benzene ring	
ACC	879	The CO_3^{2-} out-of-plane deformation vibration	
	699	The C-O in-plane deformation vibration	
	1075	The C-O stretching vibration	
	1481	The C-O antisymmetric stretching vibration	
ACC Lake	866.0	The CO_3^{2-} out-of-plane deformation vibration	
	732	The C-O in-plane deformation vibration	
	1105	The C-O stretching vibration	
	1482	The C-O antisymmetric stretching vibration	
	1643	The C=C pull-up on the benzene ring	
IC	592	Out-of-plane bending of S-O	
	1029	In-plane bending of C-H	
	1104	In-plane bending of C-H	
	1157	In-plane bending of C-H	
	1196	In-plane bending of C-H	
	1614	Stretching of C=C bonds	
	1640	Stretching of C=C bonds	

Table S3 Atomic ratios of C, N, S, Ca, Na and O on surfaces and cross-sections of particles in calcium carbonates and their colorant lakes.

Location	Sample name	C (%)	N (%)	S (%)	Ca (%)	Na (%)	O (%)
Surfaces	Calcite	11.93	0.18	0	40.76	0.55	46.58
	Calcite Lake	23.30	4.50	1.80	27.84	3.63	38.93
	Aragonite	14.77	1.26	0	44.04	0.66	39.28
	Aragonite Lake	17.70	1.44	0	31.93	0.76	48.17
	Vaterite	18.29	2.74	0	30.53	1.04	47.41
	Vaterite Lake	71.37	3.61	1.96	3.19	2.46	17.40
Cross-sections	Calcite	8.15	2.18	0	63.24	0.82	25.61
	Calcite Lake	8.15	3.34	0.02	56.64	0.99	30.85
	Aragonite	17.63	0.52	0	35.17	0.66	46.02
	Aragonite Lake	27.45	1.17	0	30.36	0.68	40.33
	Vaterite	6.63	4.40	0	66.78	0.93	21.25
	Vaterite Lake	32.66	1.14	0	41.53	0.59	24.09

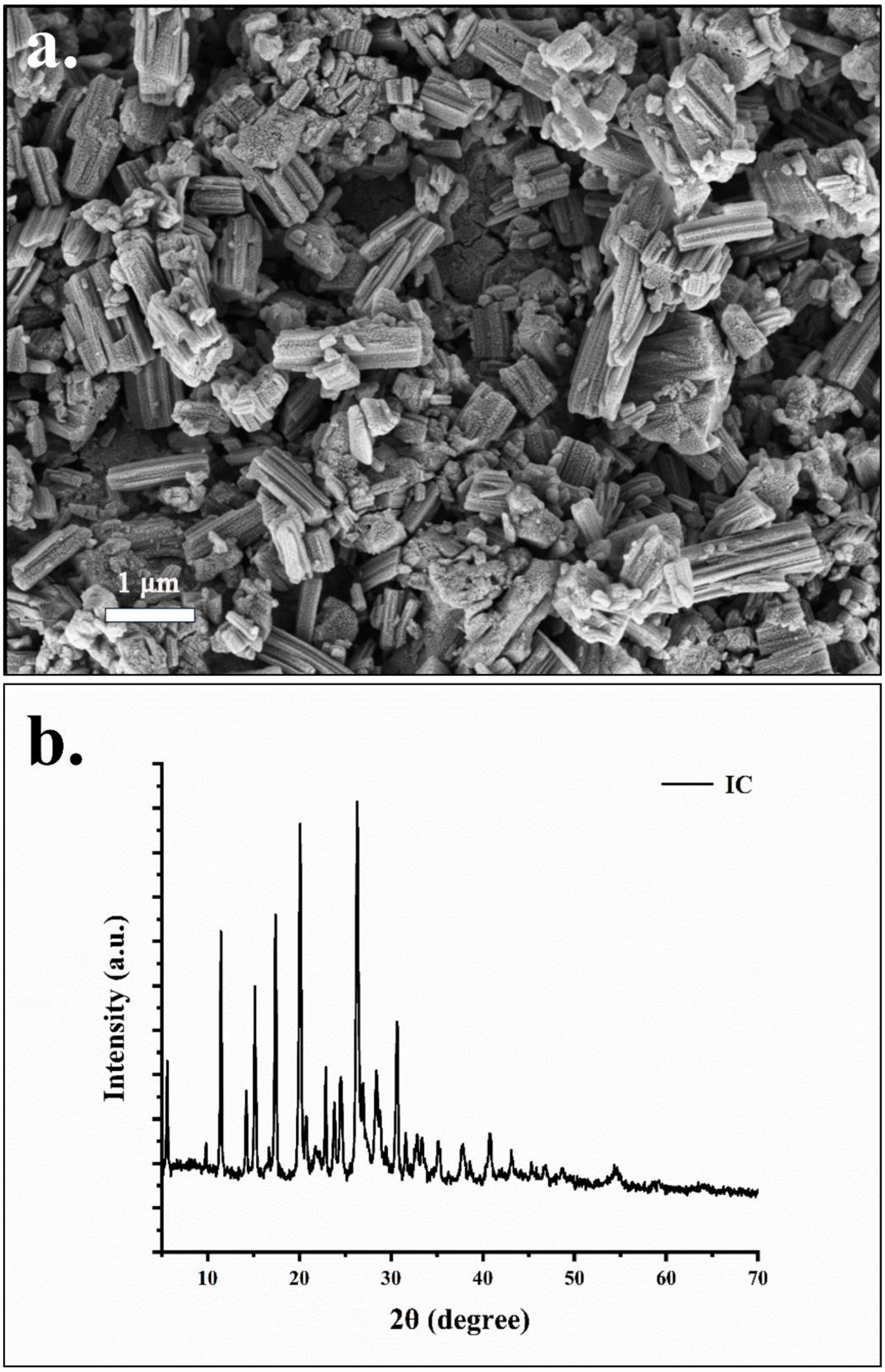

Figure S1. SEM image (a) and XRD diffractogram (b) of IC.

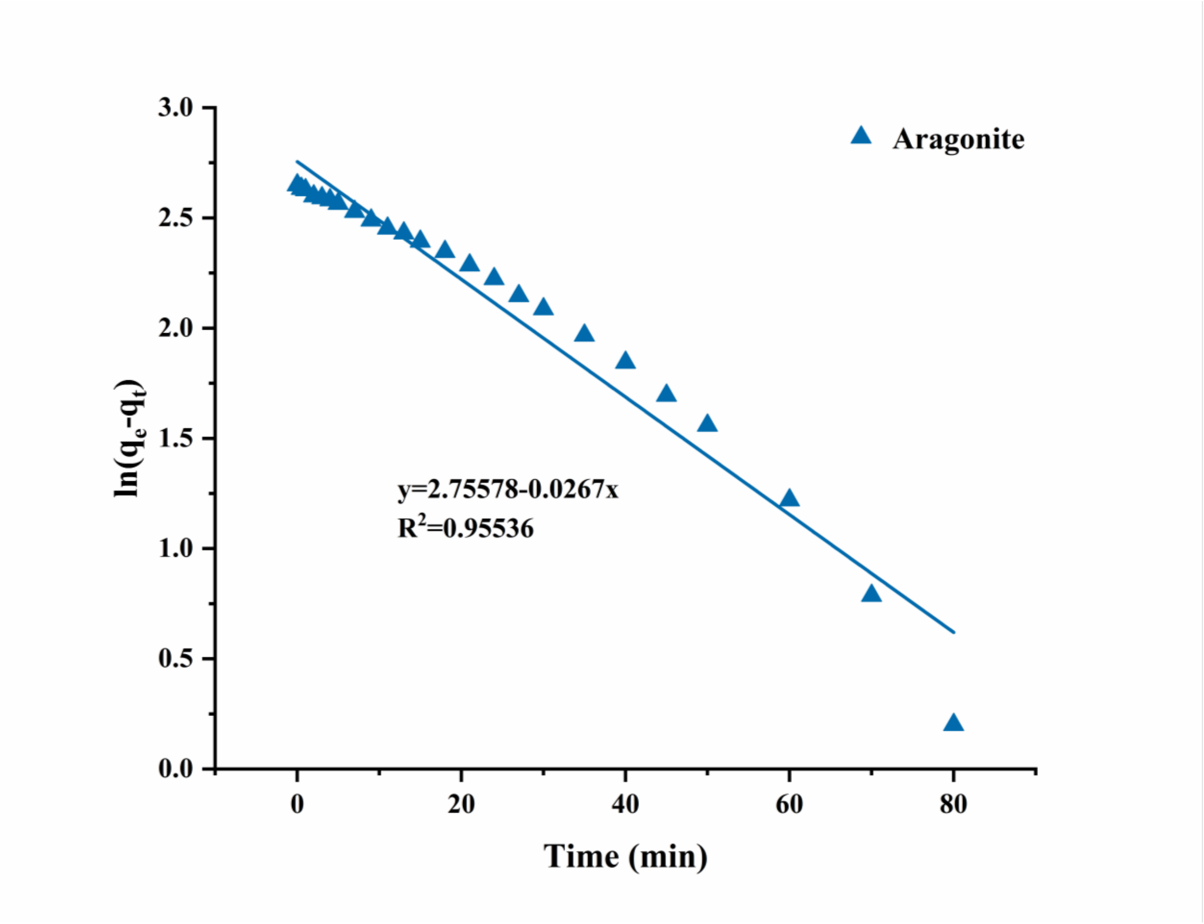

Figure S2. Pseudo-first-order kinetic model fitting curves for aragonite.

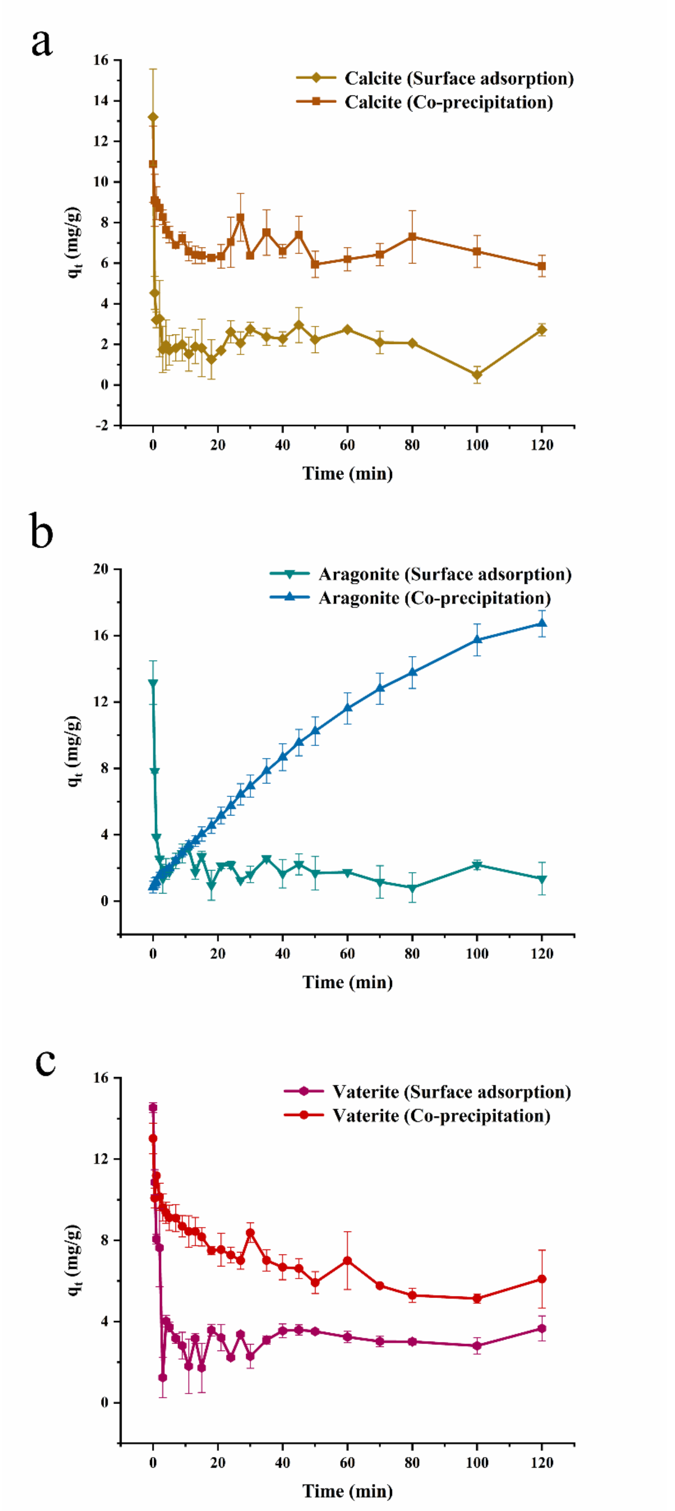

Figure S3. Kinetic curves of surfaces adsorption and co-precipitation (a: Calcite; b: Aragonite; c: Vaterite).

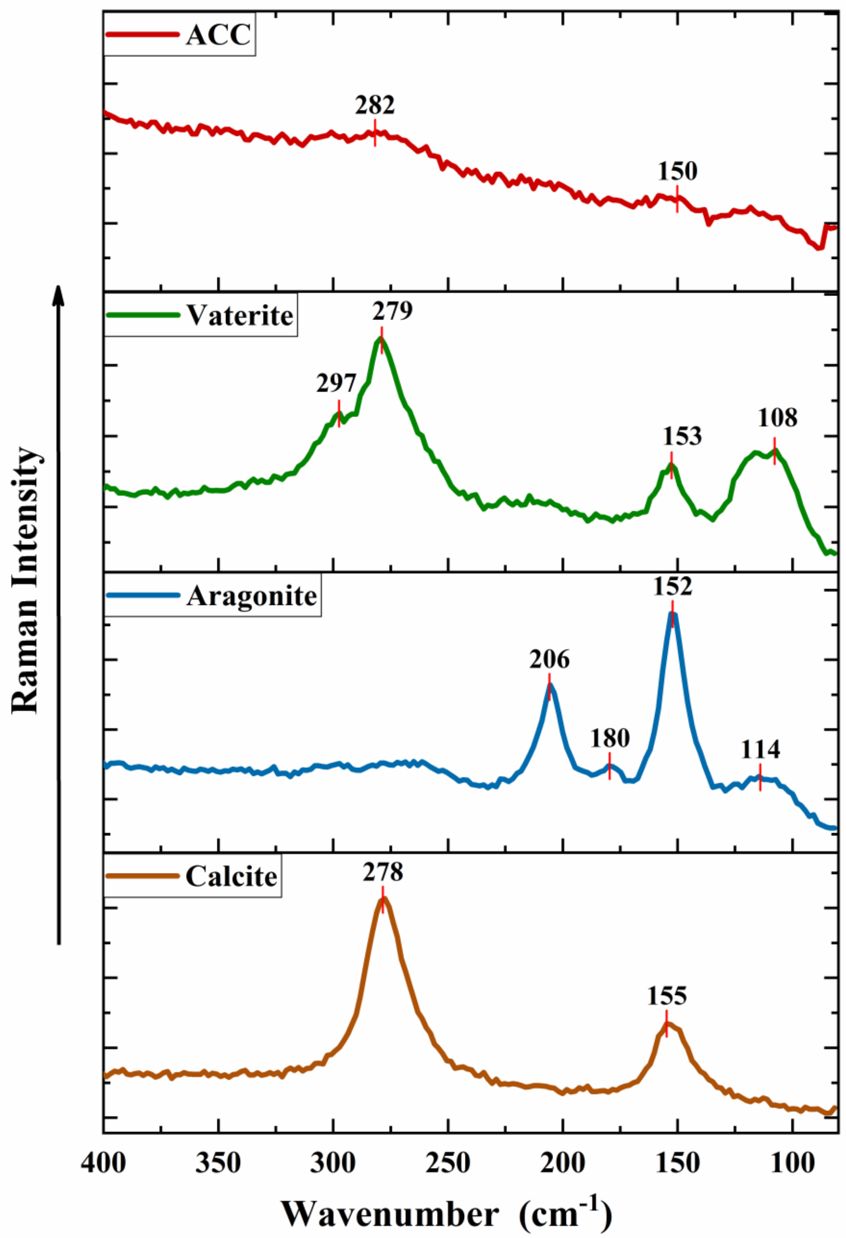

Figure S4. Lattice mode of Raman spectra of the four forms of calcium carbonate.

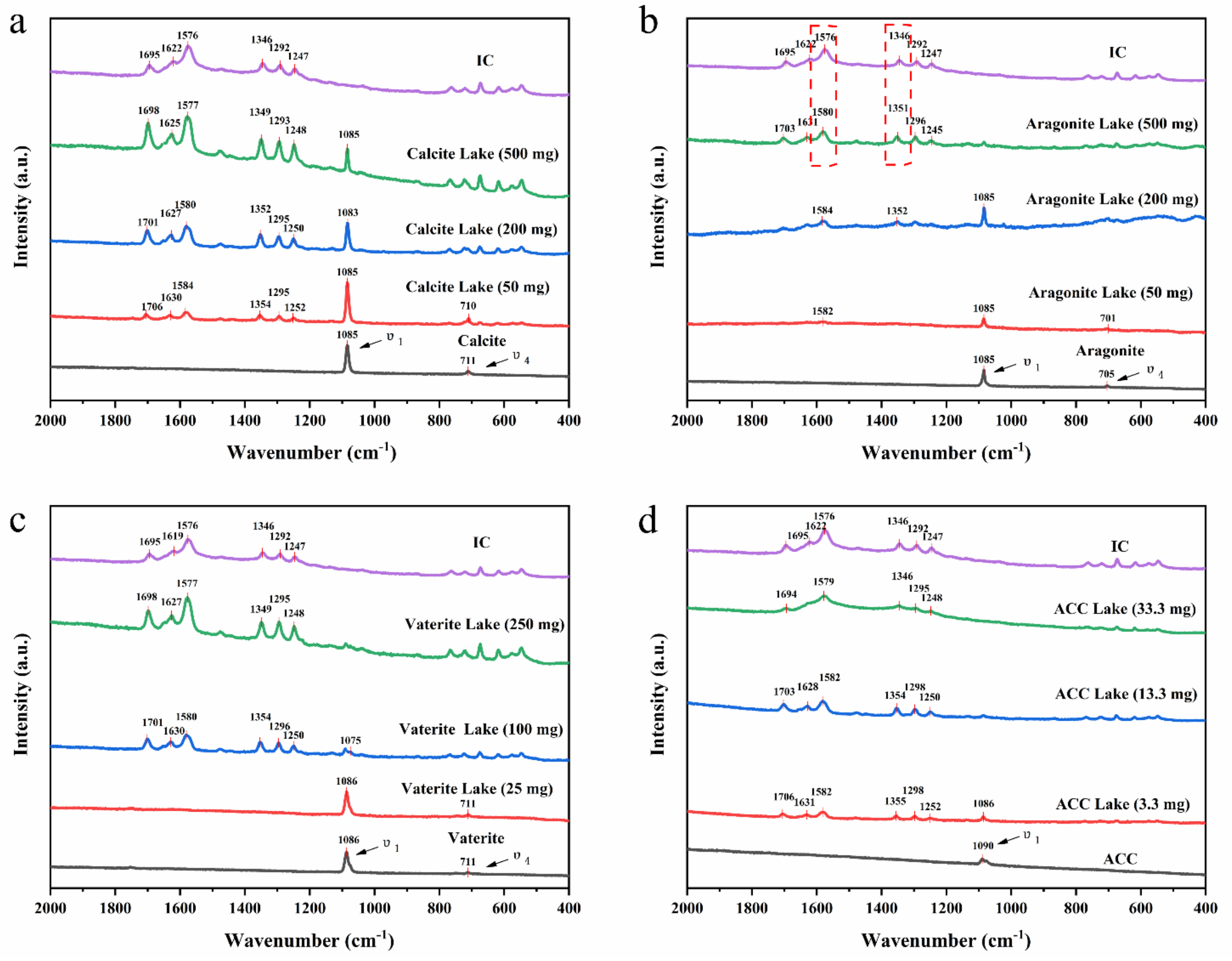

Figure S5. Internal mode of Raman spectra of the four forms of calcium carbonate.

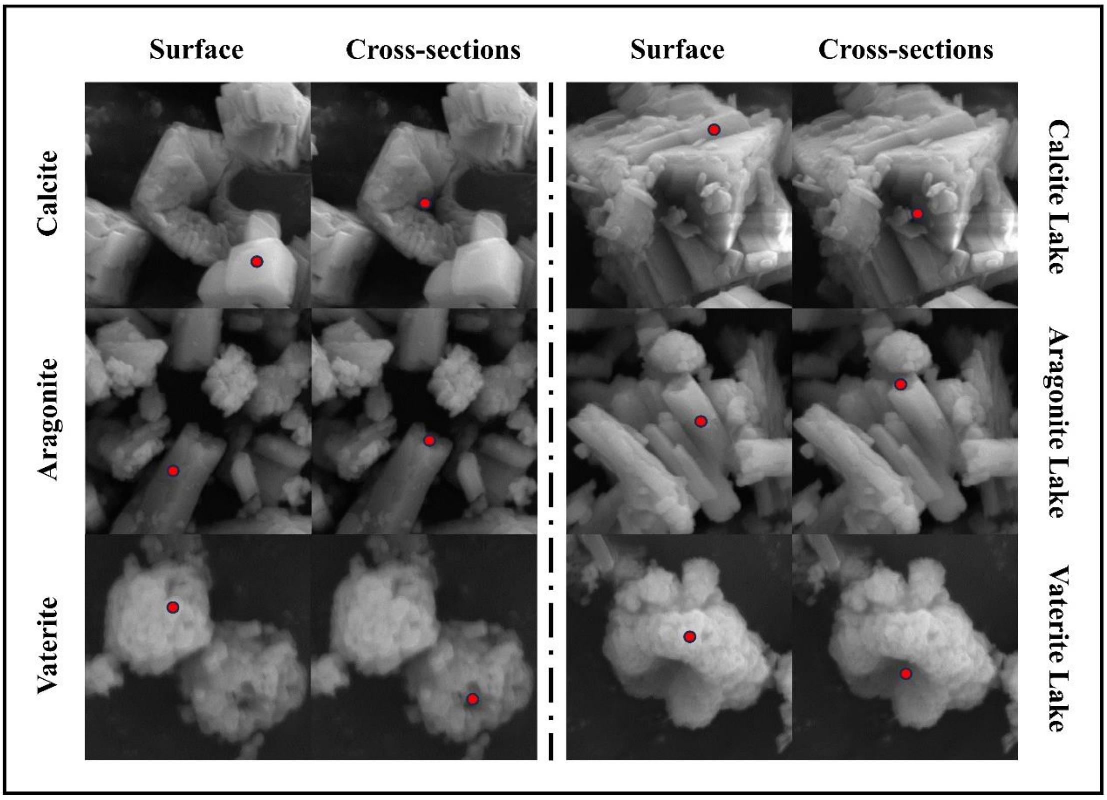

Figure S6. Representative surfaces and cross-sections of calcium carbonates and lakes (red dots are sampling points).
